# Oncological and Functional Outcomes of Transoral Robotic Surgery and Endoscopic Laryngopharyngeal Surgery for Hypopharyngeal Cancer: A Systematic Review

**DOI:** 10.3389/fsurg.2021.810581

**Published:** 2022-04-07

**Authors:** Katherine W. K. Lai, Ronald Lai, Balazs B. Lorincz, Chen-Chi Wang, Jason Y. K. Chan, David C. M. Yeung

**Affiliations:** ^1^Department of Otorhinolaryngology, Head and Neck Surgery, The Chinese University of Hong Kong, Princes of Wales Hospital, Shatin, Hong Kong SAR, China; ^2^Affiliated Teaching Unit of the Chinese University of Hong Kong, Department of Ear, Nose, and Throat, United Christian Hospital, Kwun Tong, Hong Kong SAR, China; ^3^Department of ENT, Head, and Neck Surgery, Agaplesion Frankfurter Diakonie Kliniken, Bethanien and Markus Hospitals, Frankfurt am Main, Germany; ^4^Department of Otolaryngology-Head and Neck Surgery, Taichung Veterans General Hospital, Taichung, Taiwan

**Keywords:** transoral robotic surgery, endoscopic laryngopharyngeal surgery, transoral surgery, hypopharyngeal carcinoma, laryngopharyngeal carcinoma

## Abstract

**Objectives:**

Hypopharyngeal carcinoma (HPC) is a head and neck carcinoma with poor prognosis. Traditional laryngopharyngectomy offered promising oncological outcomes at the cost of functional outcomes. The recent advent in transoral robotic surgery (TORS), an organ-preserving surgery, has opened up new perspectives in the treatment for HPC. Here, we evaluate minimally invasive organ preservation surgery [TORS and endoscopic laryngopharyngeal surgery (ELPS)] for HPC in terms of feasibility and oncological and functional outcomes.

**Methods:**

This is a systematic review. Six databases [CUHK Full-Text Journals, Embase 1910 to 2021, Ovid Emcare, Ovid MEDLINE (R), CINAHL, PubMed] were searched for articles and primary studies for TORS and ELPS for HPC. Screening was completed using predefined inclusion or exclusion criteria.

**Results:**

A total of 8 studies on TORS and 3 studies on ELPS were eventually chosen after full-text review. For studies on TORS, 61.3% of patients (84 out of 137) still survived at the last follow-up with a mean follow-up time of 23.20 months (range: 12.8–37.21 months). Severe intraoperative and postoperative complications have not been reported. No cases of TORS required a conversion to open surgery. Swallowing function was optimal postoperatively with only 6 patients eventually required a percutaneous endoscopic gastrostomy (PEG) for feeding. Disease-specific survival was taken as the parameter for the measurement of oncological outcomes. A total of 2 studies reported a disease-specific survival of 100% within their follow-up period of 1 and 1.5 years, respectively. Another 2 studies reported a 2-year DSS of 89 and 98%, respectively. A 5-year DSS of 100% in early stage and 74% in late stage were achieved in one study. Another study also reported a 5-year DSS of 91.7%. For studies of ELPS, a 5- and 3-year disease-specific survival of 100% were achieved in 2 studies. Patients who underwent ELPS had good postoperative swallowing function with no PEG placement. There were also no other fatal complications.

**Conclusions:**

Both TORS and ELPS for HPC provide satisfactory long-term oncological and functional outcomes improving postoperative quality of life of patients.

## Introduction

Hypopharyngeal carcinoma (HPC) accounts for ~6.5% of all head and neck cancers worldwide (1). Despite the low incidence, it has the worst prognosis among all types of head and neck cancers (2), due to its asymptomatic early phase and thus late presentation. In the US and Europe, it is reported that 75% of newly diagnosed patients present at stages III and IV (3), with systemic metastasis detected in 60% of patients on presentation or during the follow-up period (4). The 5-year overall survival in all stages remains at 34% in the past 20 years (1, 5). Given the poor prognosis, treatment for HPC remains a therapeutic challenge over the past decade.

Laryngopharyngectomy followed by radiotherapy with or without chemotherapy used to be the gold standard for treatment of HPC but is associated with poor functional results (6). Patients suffered from severe swallowing and speech impairment with a poor quality of life (7). Recent advancement in technology has directed efforts into developing organ-preserving surgical techniques in achieving both superior oncological and functional outcomes, giving rise to a gradual shift of treatment paradigm toward minimally invasive surgery. These less invasive transoral operations, including transoral robotic surgery (TORS) and endoscopic laryngopharyngeal surgery (ELPS), propose to offer higher functional preservation with an increasing popularity in the field of head and neck cancer (8).

The efficacy and feasibility of TORS in the management of oropharyngeal carcinoma have been well-established, demonstrating a reduction in treatment-related morbidity while maintaining comparable oncological outcomes with open surgery and primary radiochemotherapy (9). The use of TORS has been approved by the United States FDA since 2009 and currently is one of the standard treatment modalities for early oropharyngeal carcinoma (10). Whereas, most of the clinical trials studied the use of TORS in oropharyngeal carcinoma, minimal attention was given to the application of TORS in HPC due to the relatively more difficult visualization and access of hypopharynx.

Limited clinical trials are studying the long-term oncological and functional outcomes of different minimally invasive surgical techniques, namely TORS and ELPS for HPC. This systematic review aims to analyze the feasibility and efficacy of the use of TORS or ELPS as a treatment modality for HPC based on the currently available clinical trials.

## Methods

The current systematic review was conducted according to the Preferred Reporting Items for Systematic Reviews and Meta-Analyses (PRISMA) statement guidelines.

A comprehensive search strategy was created using Ovid Emcare, Embase, MEDLINE, CUHK Full-Text Journals, Cochrane database, and PubMed, searching for original studies on TORS or ELPS for hypopharyngeal malignancy published in the last 10 years (July 2012–2021). Two searches were conducted based on the types of minimally invasive surgery, one for TORS and another for ELPS. The search relied on a combination of site-specific terms (hypopharynx, hypopharyngeal, laryngopharynx, and laryngopharyngeal), disease-specific terms (carcinoma, cancer, and neoplasm), and treatment-specific terms [(transoral robotic, TORS) for TORS and (endoscopic laryngopharyngeal surgery, ELPS) for ELPS].

Articles were eligible for inclusion in the systematic review if they are original studies involving patients treated for HPC by TORS or ELPS. Only articles in the English language in the full text were considered. After a literature review of the selected databases, identical articles were removed. Articles were then screened for their relevance based on the title and abstract with irrelevant studies excluded. The remainder were read in their entirety. Articles without quantitative analysis were excluded. When studies written from the same subject pool were identified, the most complete or recent data were included.

Study information including patient demographics, surgical approach, intraoperative blood loss, oncological outcomes, and postoperative complications such as tracheostomy rates and gastrostomy tube rates was collected. Some of the studies focus on patients with pharyngeal carcinoma, which included cancers of both oropharynx and hypopharynx, from whom subsets of patients were extracted for this systematic review. Such subset analysis was conducted for studies on TORS and ELPS.

## Results

After PRISMA flow diagram on screening of clinical studies, 8 studies of TORS and 3 studies of ELPS were included in this systematic review. The list of studies is shown in Tables 1, 2. Results are as follows:

**Table 1:** Summary of the studies involved in the systematic analysis of TORS.

**Study**	**Number of patients**	**Mean follow-up (Months)**	**Recurrence**	**Overall survival**
Lörincz et al. (11)	5	17	0	80%
Durmus et al. (12)	5	13	0	100%
Wang et al. (13)	10	26	1	80%
Fujiwara et al. (14)	3	^∧^	1	^∧^
Park et al. (15)	38	60	9	45%
Mazerolle et al. (16)	57	23	7	67%
Park et al. (17)	7	^∧^	^∧^	^∧^
Hassid et al. (18)	22	37	6	55%

∧ *Data cannot be extracted*.

**Table 2:** Summary of the studies involved in the systematic analysis of ELPS.

**Study**	**Number of patients**	**Mean follow- up (Months)**	**Recurrence**	**Overall survival**
Kishimoto et al. (19)	118	47	8	93.6% (3 year) 85.5% (5 year)
Nakayama et al. (20)	8	28	0	^∧^
Kishimoto et al. (21)	13	41	1	^∧^

∧ *Data cannot be extracted*.

### Transoral Robotic Surgery

From the search results of the six databases, 102 studies on TORS were identified for potential inclusion in this systematic review. With duplicates removed, 38 studies remained. These studies were screened by the title, abstract, and part of the contents for irrelevant studies to be removed. Subsequently, 14 studies were identified as potentially meeting the inclusion and exclusion criteria. After thorough analysis, 8 studies were included in this review.

**Figure 1:**
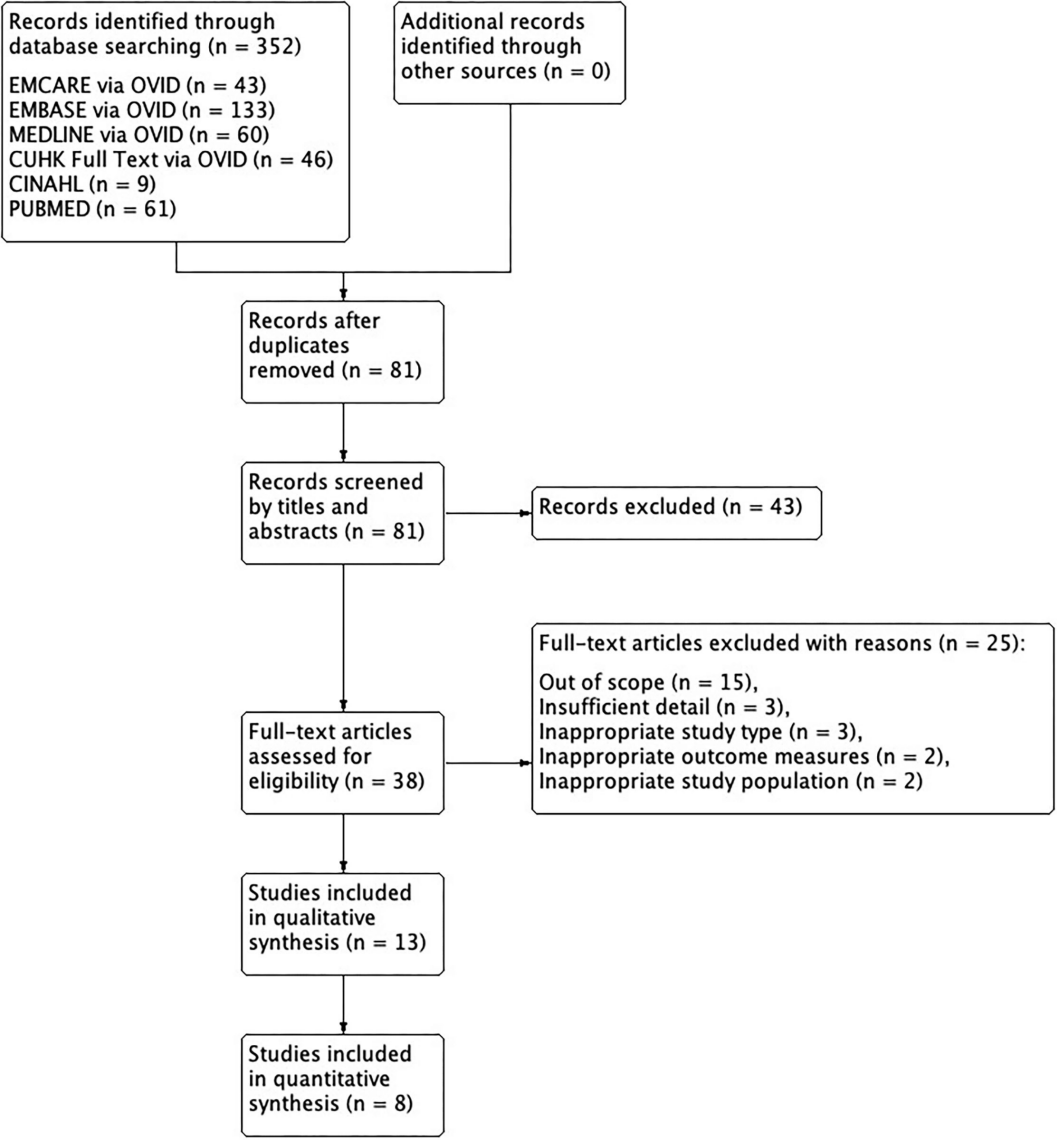
PRISMA flow diagram on TORS.

Among the 8 studies, 6 recruited patients with HPC alone, 1 studied specifically pyriform sinus carcinoma and 1 included patients suffering from pharyngeal cancers. No additional studies were identified from the reference lists of the included studies (Figure 1).

### Endoscopic Laryngopharyngeal Surgery

From the search results of the six databases, 25 studies on ELPS were identified for potential inclusion. With duplicates removed, 24 studies remained. A number of 18 studies were identified as potentially meeting the inclusion and exclusion criteria after those irrelevant were screened. After analysis, 3 studies were eventually included in this review after detailed analysis.

**Figure 2:**
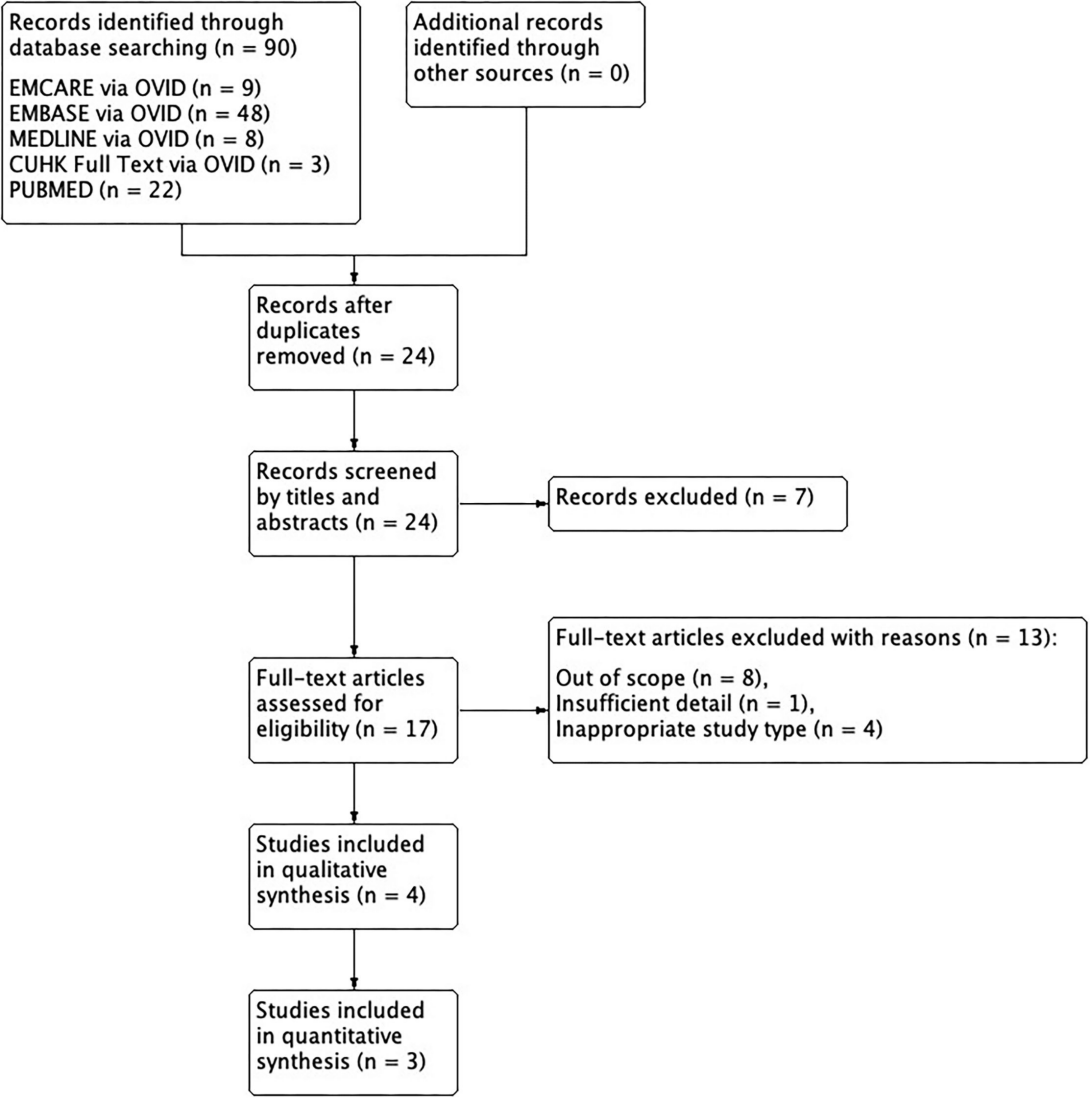
PRISMA flow diagram on ELPS.

All of these studies focused on HPC, with 1 study that is focused on the elderly populations. No additional studies were identified from the reference lists of the included studies (Figure 2).

### Study Characteristics

Transoral robotic surgery studies were conducted in France, Germany, Japan, South Korea, Taiwan, and the United States of America, whereas all ELPS studies selected were from Japan. Their publication dates range from 2013 to 2020. A total of 4 prospective studies and 4 retrospective primary studies were included for TORS, whereas for ELPS, all studies were retrospective. None of these studies compared their case groups with a control group.

### Synthesis of Results

#### Transoral Robotic Surgery

##### Demographics and Disease State

A total of 8 studies on TORS were included in this systematic review. A total of 147 patients presenting with HPC were included, with a mean age of 61.69 (59.8–66.7). Alcohol usage and smoking status were reported in 3 of the selected studies which evaluated a total of 84 patients, among whom 64 patients were drinkers and 74 patients were smokers.

Among the 147 patients, the vast majority, 121 of the patients, had tumors arising from the pyriform fossa, 14 patients with tumors at the posterior pharyngeal wall, 3 from the aryepiglottic fold, 1 from the postcricoid, and 1 from the lateral pharyngeal wall. A number of 2 of these tumors were reported to be p16-positive and 1 of these was HPV-DNA-positive.

All patients were diagnosed with squamous cell type of HPC, except 1 who presented with undifferentiated cell type. For patients with squamous cell carcinoma, their T staging was as follows: 62 patients classified as T1, 59 as T2, 13 as T3, and 6 as T4. Seven patients' T statuses were not available. Thirty-eight patients were staged as stage 1, 20 stage 2, 21 stage 3, and 51 stage 4 (Table 3).

**Table 3:** Patient and lesion characteristics of TORS.

**Patient characteristics**	
Number of patients	147
Number of lesions	147
Age	61.69 (59.8–66.7)
**Lesion characteristics**	
Subsites	
Pyriform fossa	121
Postcricoid	1
Posterior pharyngeal wall	1
Lateral pharyngeal wall	14
Aryepiglottic fold	3
**T staging**	
T1	62
T2	59
T3	13
T4	6

##### Treatment of the Primary Lesion

Transoral robotic surgery was performed for patients with HPC under general anesthesia with the use of endoscopes, dissectors, and retractors, which were specified in 7 studies. Two retractor systems were used in these studies, the Laryngeal Advanced Retractor System (LARS) by Fentex Medical and the Feyh-Kastenbauer oropharyngeal retractor modified by Weinstein-O' Malley (FK) by Olympus-Gyrus. A number of 4 studies used FK only, 1 used LARS only, and 2 used both retractor systems. The robotic-assisted resection time varies from 44 to 93 min from 3 studies, with a mean of 70.63 min.

Ipsilateral or bilateral neck dissections were performed in 104 out of 140 (74.2%) patients, and majority of the patients with level II to IV lymph nodes were removed.

Neoadjuvant chemotherapy was given in the study of Park et al. for 10 out of 38 patients, 6 for T4 disease and 4 for T3 nodal disease, with either partial or complete response. The remaining studies did not report the use of neoadjuvant therapy. Adjuvant chemoradiotherapy or radiotherapy was given in selected patients with HPC in the presence of multiple positive lymph nodes, positive resection margins, tumors with certain histopathology, or extracapsular or lymphovascular invasion. A number of 91 out of 147 (61.9%) patients were offered adjuvant therapy, 44 with chemoradiotherapy, and 45 with adjuvant radiotherapy. Radiation dose varied among individuals, with the average dose of 57.2, 60, 66, and 66.3 Gy reported in 4 studies. Two patients received another pharyngolaryngectomy as salvage surgery.

##### Intra- and Postoperative Outcomes

Of the 147 patients who underwent TORS, 1 study demonstrated 4 out of 5 patients with surgical margins achieving 5 mm or more and 1 patient with a surgical margin of 4 mm. In the other 7 studies, positive surgical margins were reported in 24 patients, close margin in 1 patient, and negative surgical margins in the rest after reexcisions. Overall, negative margins were achieved with TORS in 122 out of 147 patients (83.0%) and positive margins in 24 patients (16.9%). No conversion to open surgery was reported across all studies.

**Figure 3:**
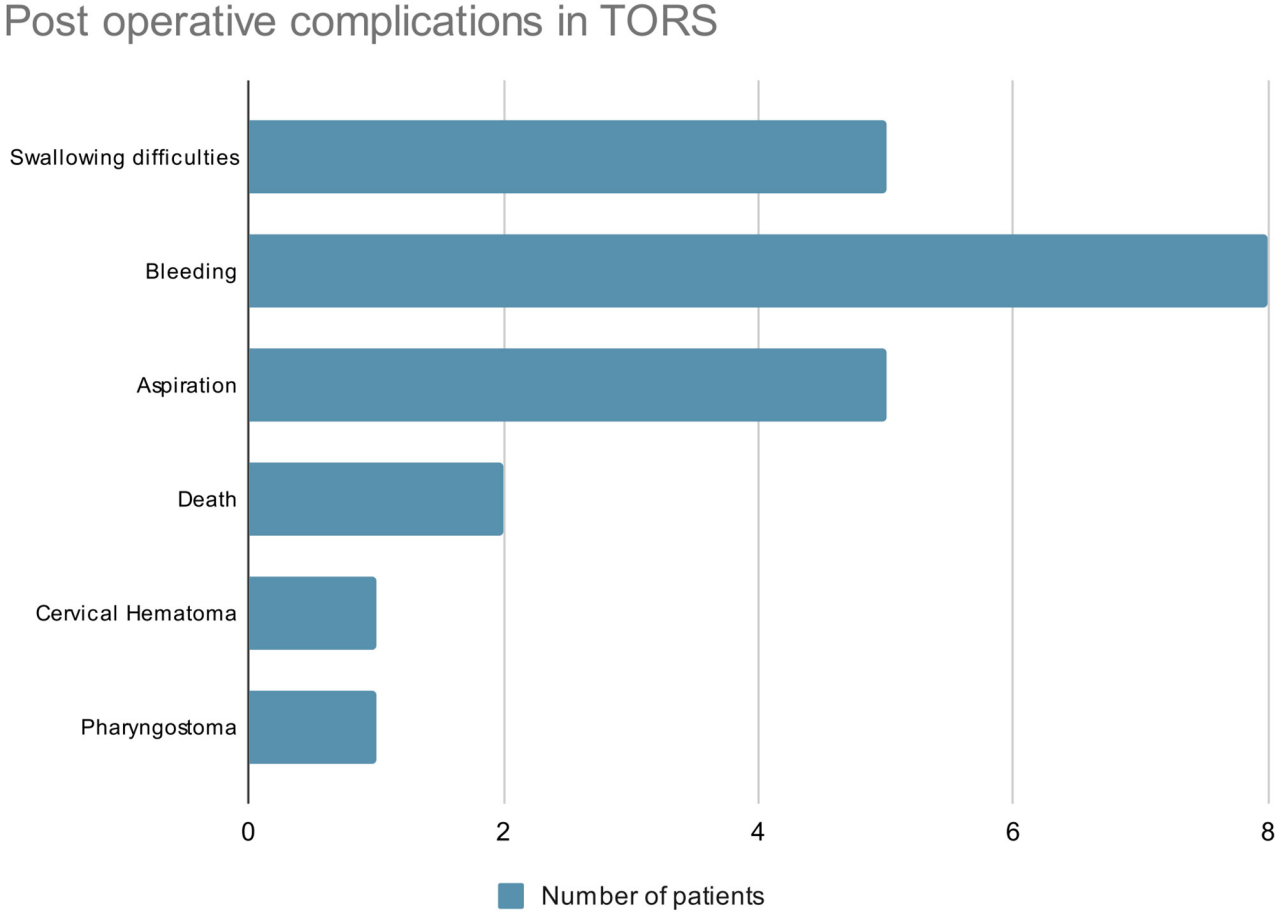
Post-operative complications in TORS.

An average blood loss of 55 mL was reported in 1 of the studies. No severe blood loss or transfusion requirement was reported. In the perioperative period, 57 elective tracheostomies (38.8%) were performed in 5 studies as prophylactic airway protection. Other postoperative complications that included 5 aspiration pneumonia reported from 2 studies and postoperative bleeding in 8 cases. In the French GETTEC group study, 1 cervical haematoma, 1 pharyngostoma, and 2 deaths were reported (Figure 3).

Swallowing and feeding functions were evaluated. Functional outcome swallowing score (FOSS) was taken as the parameter for measurement in 3 studies. A mean FOSS of 0.8 was reported in 1 study whereas 29 patients (76.3%) achieved FOSS 0–2 in another study. In a study conducted in Japan, all 3 patients achieved FOSS 0–1. The other 3 studies reported no adverse event related to aspiration. There was 1 report of transient dysphagia with a return to normal swallowing in 3 months. Only 1 study reported transient laryngeal aspiration in 16 patients (30%) with 2 patients eventually developing pneumonia. Overall, feeding routes of 6 patients converted from nasogastric tubes to PEG tubes for nutritional support with 1 case due to adjuvant chemoradiotherapy, 2 due to adjuvant radiotherapy, and 1 postoperative dysphagia. Five of these were listed as transient PEG with unreported durations.

Speech functions post-TORS were assessed in 3 studies. The number of 1 study reported a mean Voice Handicap Index−10 of 9.6; another study reported the return of speech functions in 3 months. Only 1 study reported increased jitters on acoustic waveform analysis.

##### Oncological Outcomes

**Figure 4:**
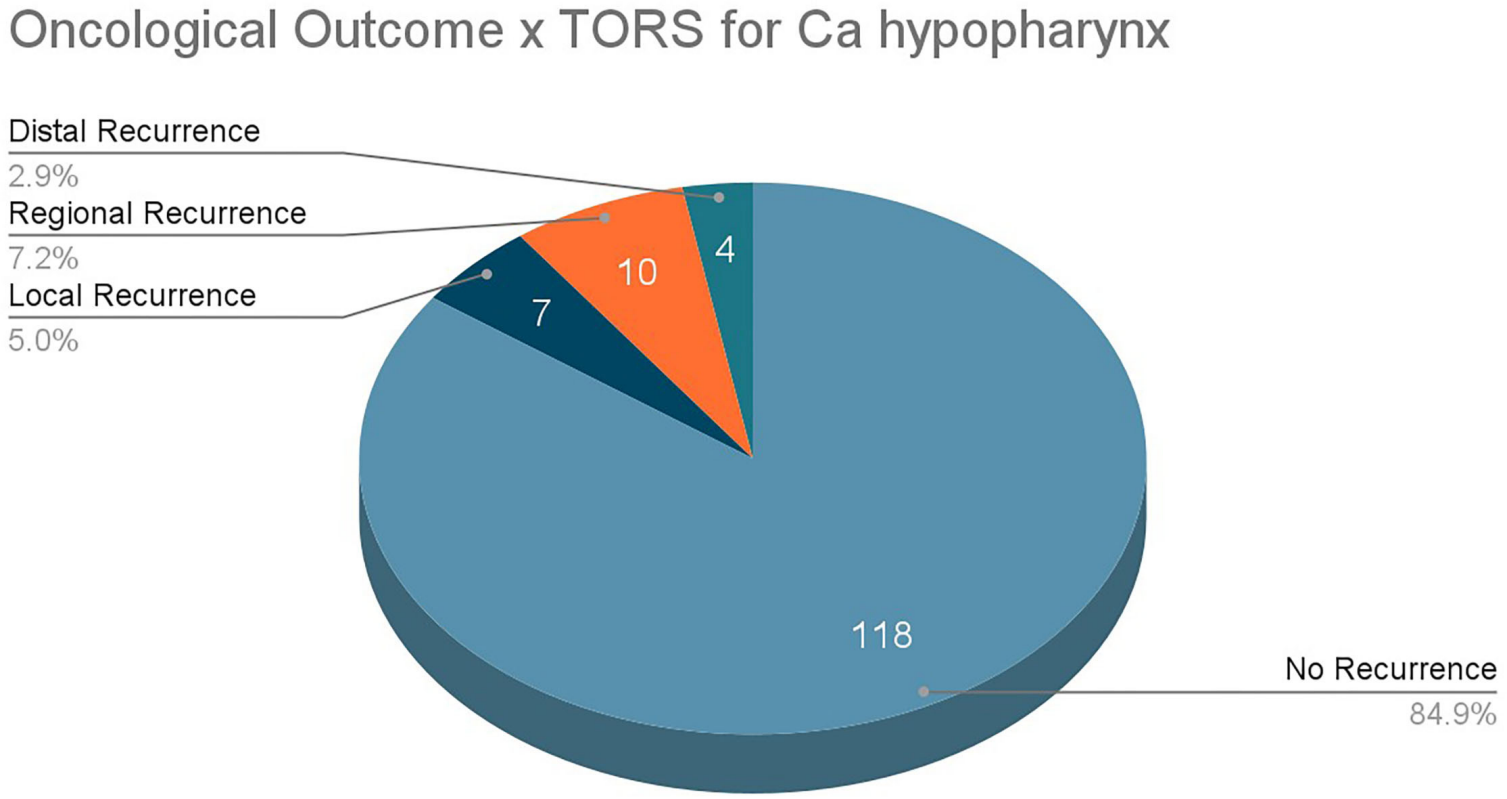
Oncological outcomes in TORS patients.

All patients were followed up regularly after TORS, and the follow-up durations range from 12.8 to 37. Twenty one months with an average of 23.20 months. There were 7 patients (5.00%) experiencing local recurrence during the follow-up period, 10 patients (7.20%) with regional recurrence, and 4 patients (2.90%) suffering from distant metastasis (Figure 4).

**Figure 5:**
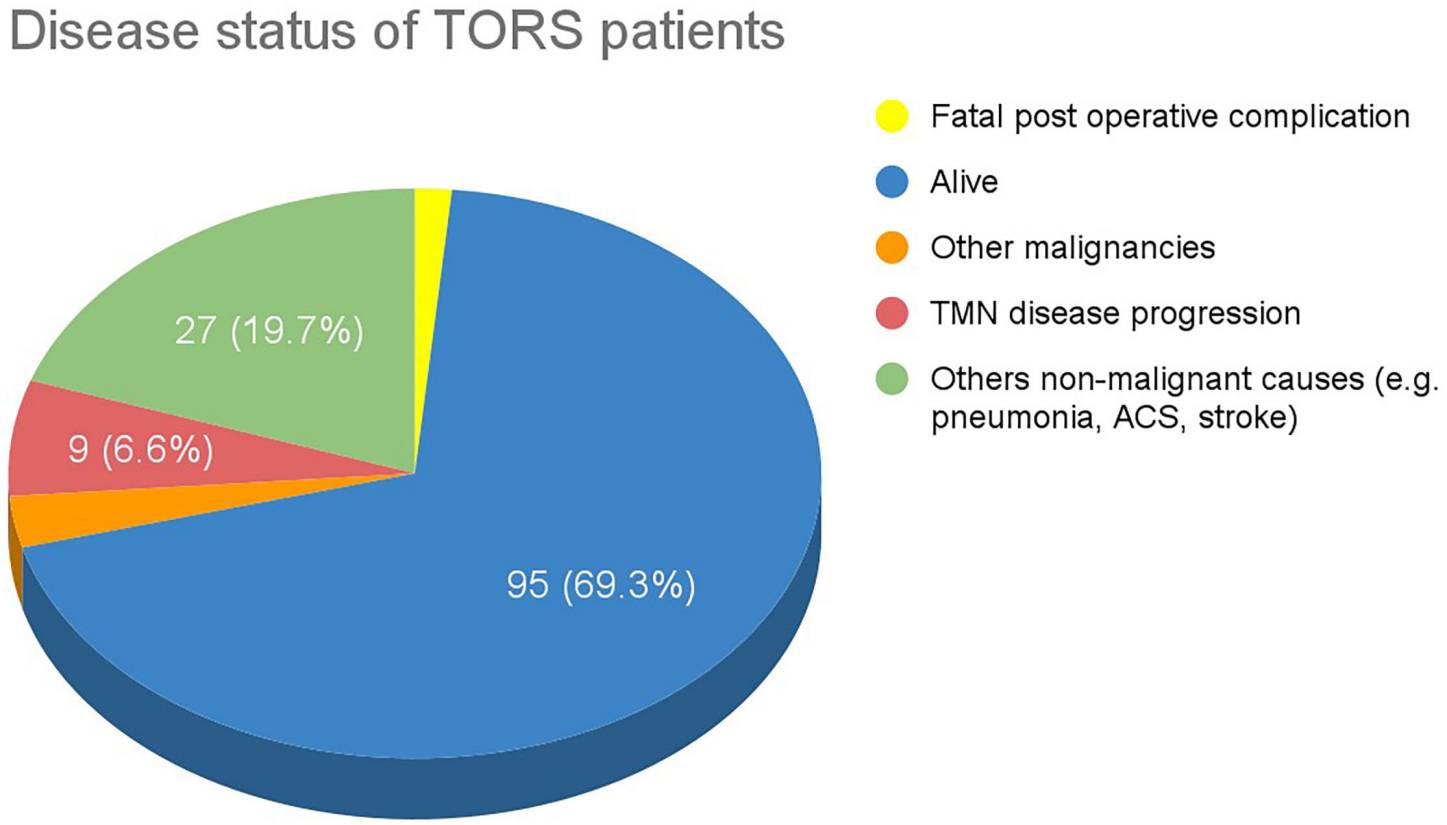
Disease status of TORS patients.

Of the 137 patients reported from 6 studies, 95 (69.3%) were alive at the final follow-up, with a mean follow-up time of 23.20 months and a range of 12.8 to 37.21 months. A total of 9 patients (6.60%) died from disease progression and 27 died from other causes. Two patients among the 137 patients reported died of postoperative complications, both presenting with advanced cirrhosis and, respectively, suffering from multiorgan failure and recurrent blood loss of unknown source (Figure 5).

During the follow-up period, the disease-free survival of stage I to II diseases was 67.2% (39 out of 58 patients) as reported from 4 studies. Another study of the French GETTEC group reported a 74 and 50% disease-free survival at 24 and 48 months, respectively, in all stages of the disease. The Hassid 2020 study reported an overall 5-year disease-free survival of 57.1%.

#### Endoscopic Laryngopharyngeal Surgery

##### Demographics and Disease State

Three studies on ELPS were included in this systematic review. In one of the studies selected, “A Clinical Study of Transoral Pharyngectomies to Treat Superficial Hypopharyngeal Cancers,” additional endoscopic approaches to superficial HPC were included in the primary study, namely endoscopic mucosal resection and endoscopic submucosal dissection. For this particular study, only data of ELPS were included. A total of 139 patients diagnosed with HPC were included, with a mean age of 70.65, ranging from 65.60 to 79.15. Only one study reported the drinking and smoking statuses of its study population, with 114 patients out of 118 being drinkers and 103 smokers.

Some patients included in this systematic review presented with multiple hypopharyngeal lesions, and thus, 179 hypopharyngeal lesions from 139 patients were included in this review. Outcome measures from one of the studies cannot be extracted, and thus, 171 lesions were eventually included. The majority of these lesions arise from the pyriform fossa, accounting for 130 lesions (76.0%), 29 lesions from postcricoid, followed by 12 from the posterior pharyngeal wall (Table 4).

**Table 4:** Patient and lesion characteristics of ELPS.

**Patient characteristics**	
Number of patients	161
Number of lesions	207
Age	70.65 (41–85)
**Lesion characteristics**	
Subsites	
Pyriform fossa	152
Postcricoid	32
Posterior pharyngeal wall	0
Lateral pharyngeal wall	23
Aryepiglottic fold	0
**T staging**	
Tis	42
T1	45
T2	22
T3	11
T4	0
Others (data not isolated)	35

Data of a total of 171 precancerous and cancerous lesions were extracted and included. A total of 42 of them were tumor *in situ* whereas the remaining 129 hypopharyngeal lesions were cancerous with 45 tumors graded as T1, 22 were T2, 11 were T3, and no tumor reported as T4. The regional and systemic staging was not available in these studies (Table 4).

##### Treatment of the Primary Lesion

Endoscopic laryngopharyngeal surgery was performed under general anesthesia with the use of curved rigid pharyngolaryngoscopes. In 1 of the 3 studies analyzed, multiple lesions will be removed in the same operation with no more than 4 lesions removed each time. Thus, only 163 lesions were resected among the 171 hypopharyngeal lesions found. Neck dissections were performed in selected cases where cervical lymph node metastasis was evident, and level of lymph nodes was not mentioned.

No neoadjuvant therapy was given in any of the studies. Postoperatively, 2 patients underwent adjuvant chemoradiotherapy due to the presence of cervical lymph node metastasis.

##### Intra- and Postoperative Outcomes

Of the 163 hypopharyngeal lesions that were treated with ELPS, 1- to 2-mm resection margins were taken in one of the studies accounting for 142 resections. Among 160 surgical specimens with surgical margins reported, positive margins were identified in 26 specimens. In addition, 11 specimens were reported to have uncertain surgical margins due to damages to the edges of the surgical specimen during ELPS. One of the studies which reported 20 positive surgical margins concluded that there was no correlation between positive surgical margins and the risk of recurrence. None of the studies reported conversion to open surgery.

Among all patients who underwent ELPS, only 3 patients (2.16%) required tracheostomies, 2 of which performed for postoperative bleeding and none permanent. All patients regained swallowing function postoperatively and resumed oral intake, with the average oral fasting periods reported as 4.4, 4.9, and 5.6 days from the 3 studies. The shortest fasting period was 1 day whereas the longest observed was 78 days. No PEG tube dependency was described. No vocal fold impairment was observed.

**Figure 6:**
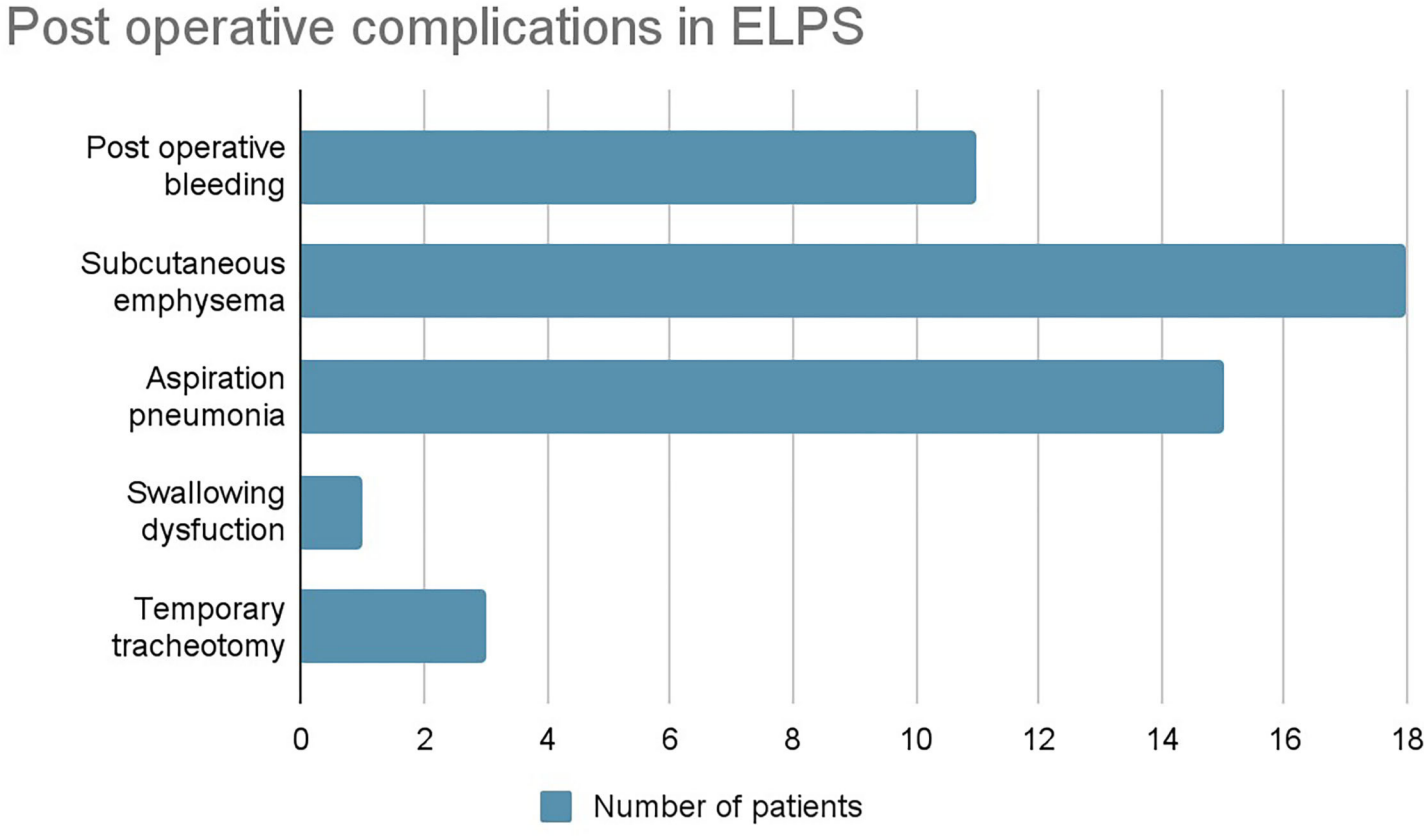
Post-operative complications in ELPS.

Other postoperative complications were postoperative bleeding in 11 patients, subcutaneous emphysema in 18, aspiration pneumonia in 15, and temporary swallowing dysfunction in 1 patient who eventually recovered (Figure 6). Lengths of in-patient stays were reported in 2 studies, with an average of 14.4 days. No mortalities resulted from these complications.

##### Oncological Outcomes

**Figure 7:**
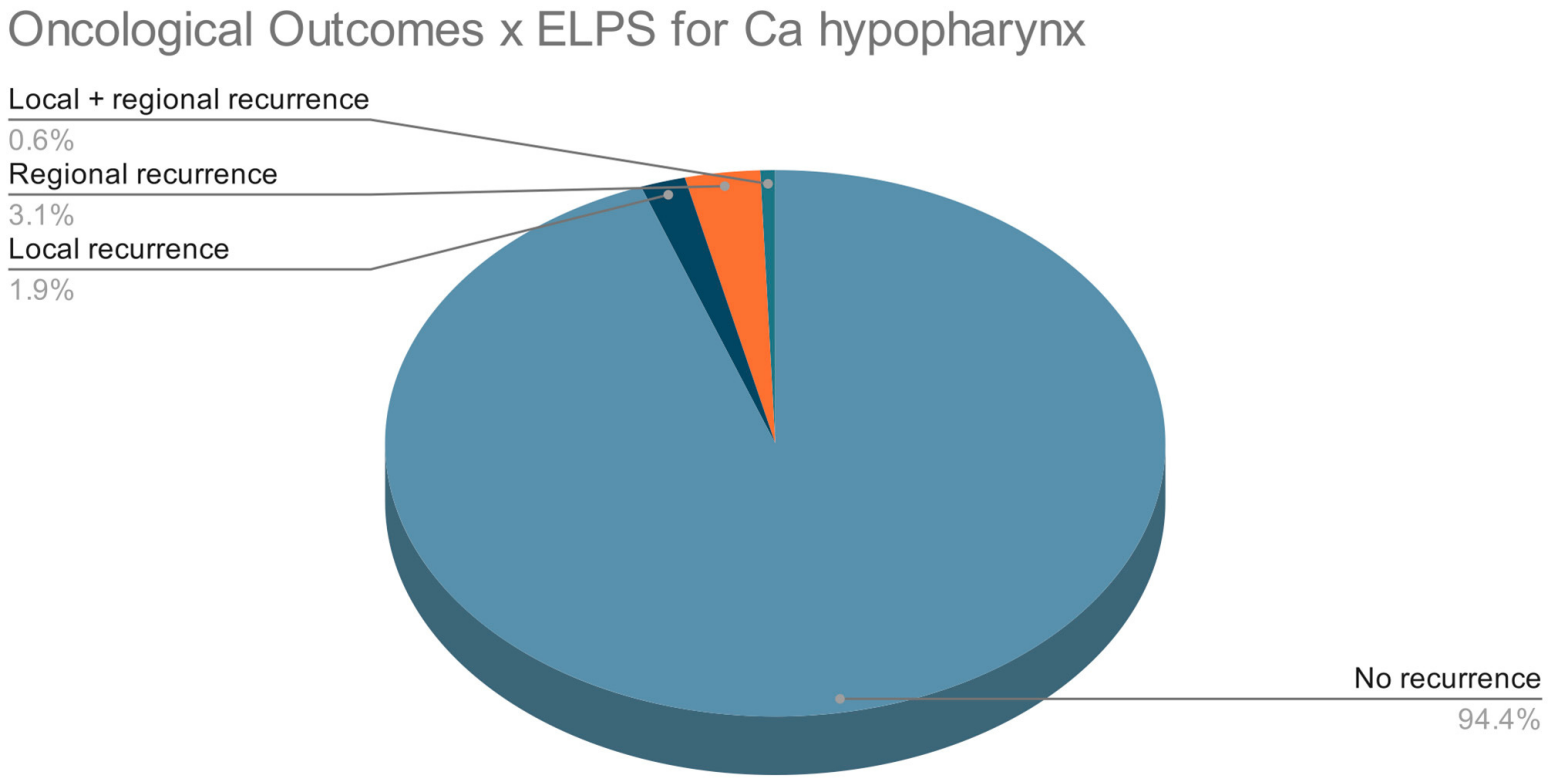
Oncological outcomes in ELPS patients.

All patients were followed up regularly post-ELPS with an average follow-up duration of 44.0 months from the 3 selected studies. The number of 3 patients experienced local recurrence during the follow-up period, 5 patients regional recurrence, whereas 1 patient suffered from both local and regional recurrence. No distant metastasis was reported in all 139 patients (Figure 7).

Only 1 of the 3 selected studies in this systematic review specifically reported the overall survival rate of patients diagnosed with HPC who underwent ELPS. The remaining 2 studies include patients with oropharyngeal carcinoma treated with ELPS or HPC treated with other endoscopic means. The overall survival and disease-specific survival rates from these 2 studies cannot be isolated.

**Figure 8:**
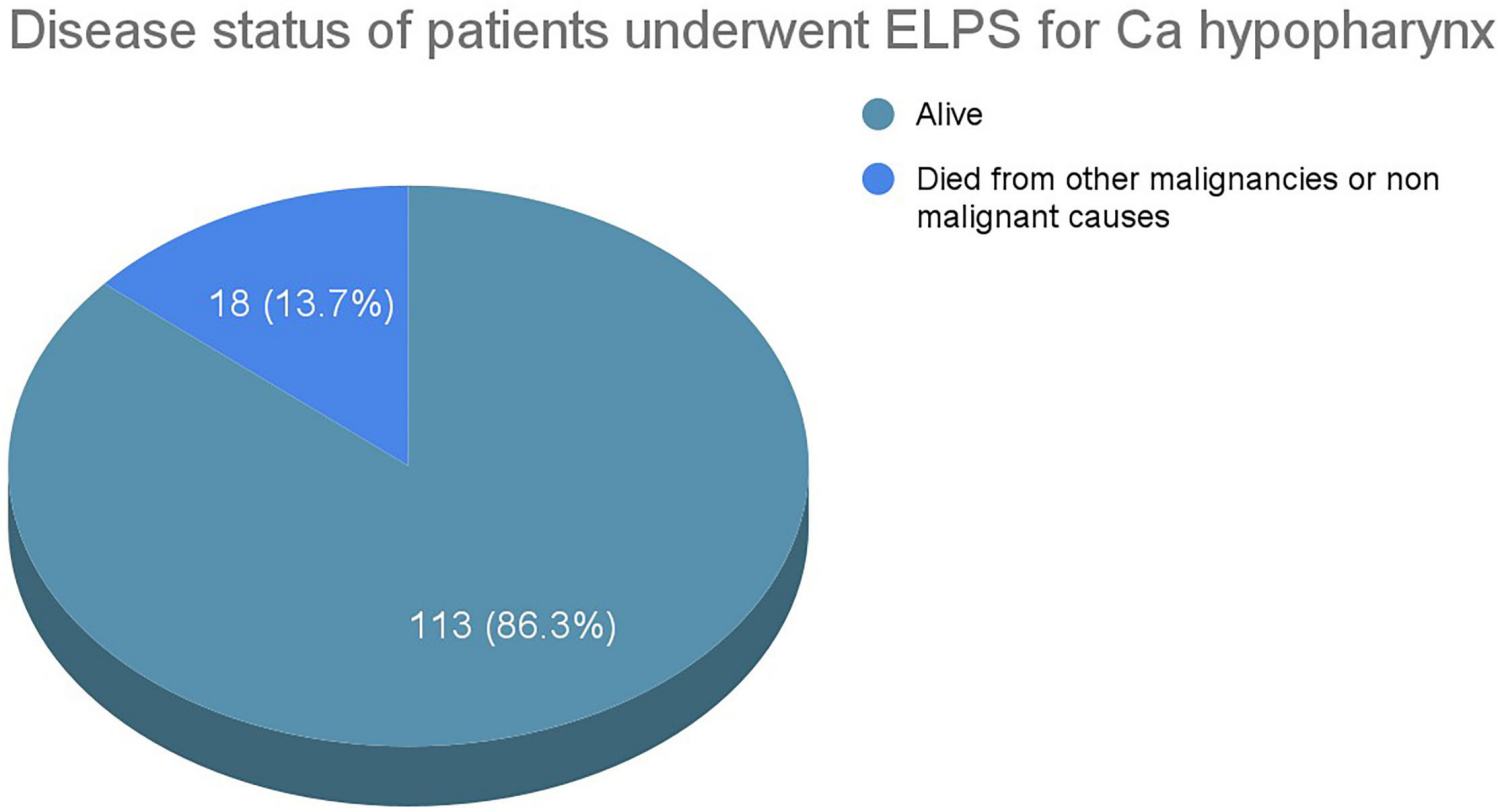
Disease status of ELPS patients.

From the study with patients with exclusively HPC treated with ELPS, 16 out of 118 patients died from other unrelated causes, such as other primary malignancies or pneumonia. The rest of the patients survived till the last follow-up with the mean follow-up duration of 44 months (Figure 8).

#### Comparison Between TORS and ELPS

Both TORS and ELPS are acceptable treatment modalities for HPC. When considering which treatment modality to use, the oncological and functional outcomes should be weighed accordingly.

**Figure 9:**
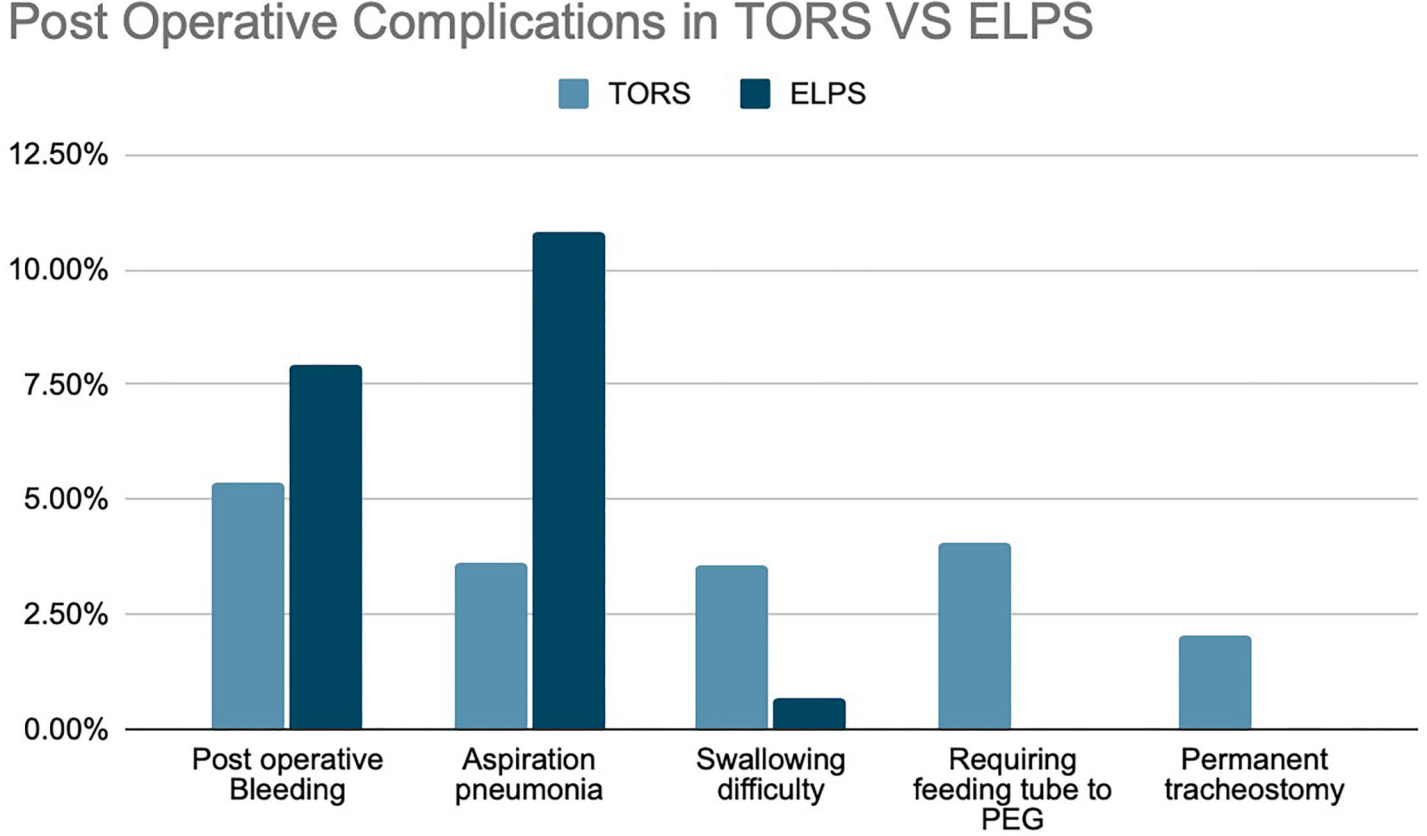
Post-operative complications between TORS and ELPS.

This systematic review examines the perioperative and postoperative complications of patients who underwent TORS and ELPS (Figure 9). Complications of 147 TORS patients and 139 ELPS patients were compared using chi- square test and Fisher's exact test.

A chi-square test of independence was performed to examine the relationship between treatment modality and 3 of the postoperative complications, namely postoperative bleeding, aspiration pneumonia, and swallowing difficulty. *p*-Values are as follows: postoperative bleeding, *p* = 0.70; aspiration pneumonia, *p* = 0.01; swallowing difficulty, *p* = 0.12 (Table 5). Statistically significant difference was shown in aspiration pneumonia.

**Table 5:** Comparison of different complications between TORS and ELPS.

**Variable**	***p*-value**
Postoperative bleeding	0.70
Aspiration pneumonia	0.01*
Swallowing difficulty	0.12
Permanent tracheostomy	0.25
Feeding tube to PEG	0.03*

* *Significant difference with p < 0.05*.

Fisher's exact test was performed for 2 other variables, the need for permanent tracheostomy and PEG feeding. No significant relation was established for permanent tracheostomy with *p* = 0.25. The relation between treatment modality (i.e., TORS VS. ELPS) and the need for PEG was significant, *p* = 0.03 (Table 5). Patients who underwent TORS are more likely than ELPS to require conversion of the feeding tube to PEG feeding.

Comparison of oncological outcomes between TORS and ELPS was not performed in this systematic review due to the difference in the mean follow-up period in the studies recruited. The variation in the parameters used for the measurement of oncological outcomes also made direct comparison challenging in this case.

## Discussions

The treatment strategies for HPC have evolved over the years. There is still no level-one evidence on the best treatment nor universal consensus regarding the optimal treatment strategy (22). A combination of surgery and radiotherapy was once the mainstay of treatment. In one of the largest studies conducted from 1990 to 1992 with 2,932 patients recruited in the USA, patients were treated with surgery with or without radiotherapy, radiotherapy alone, or chemoradiotherapy. The 5-year disease-specific survival was 33.4% without significant variation between different treatment modalities (13), with similar findings concluded from another study from the Netherlands (23). The poor functional outcome and reconstruction complications associated with surgical resection have led to the popularity of organ preservation chemoradiotherapy for the treatment of laryngopharyngeal carcinoma. The 2-year disease-specific survival rates were 55 and 41% in 2 individual studies (24, 25). However, organ preservation chemoradiotherapy is still associated with poor functional outcomes, with studies reporting patients depending on permanent tracheostomy and tube feeding due to impaired swallowing function (26).

With the advancements in technology, minimally invasive surgery has gathered attention because of its limited tissue dissection and good functional outcomes. Whereas, TORS has been widely applied in oropharyngeal carcinoma (27), only a few studies reported the use of TORS in HPC. Thus, in this systematic review, we evaluated the oncological and functional outcomes of TORS and ELPS for Ca hypopharynx.

Only a total of 8 primary studies with 147 patients and 3 studies with 139 patients were available for review for TORS and ELPS, respectively. To validate these new treatment modalities curative intent, oncological clearance must be emphasized. TORS for the treatment of HPC has shown promising results, with 84.9% of patients experiencing no recurrence in the follow-up periods. A total of 69.3% of TORS patients survived throughout the follow-up periods whereas 22.6% died from other malignancies or non-malignant causes. Studies from ELPS also illustrated good oncological outcomes where 94.4% of patients had no recurrence of the disease. A total of 86.3% of the post-ELPS patients were alive at the end of the follow-up period whereas the remaining 13.7% of patients died from intercurrent illnesses with none of these patients succumbing to disease progression of HPC.

Functional outcomes and postoperative quality of life are some of the major gains of minimally invasive surgery over conventional approaches. In these studies, both TORS and ELPS demonstrated encouraging results in the functional aspects of life. Only 5 patients who underwent TORS experienced dysphagia postoperatively that necessitated enteral tube feeding or PEG. Besides, all non-oral feeding was transient and patients eventually resumed oral intake. In ELPS, transient dysphagia was reported in only 1 patient. The minimally invasive approaches also avoided post-reconstruction complications such as flap failure or anastomotic leak which would impair the rehabilitation and quality of life of these patients.

The need for tracheostomy is not uncommon following traditional laryngopharyngectomy or chemoradiotherapy. Not only does it impair speech production, tracheostomy is also associated with complications such as tracheal stenosis, malacia, and fistula formation. This systematic review has illustrated that the use of minimally invasive approaches—TORS and ELPS—can effectively reduce the need for tracheostomy. Only 57 TORS patients and 3 ELPS patients eventually required a perioperative tracheostomy, 3 of which were permanent. The necessity of perioperative tracheostomy for access issues can be further avoided with the application of the latest Evone system from Ventinova. It is a closed ventilation system with a thin cuffed tube. It utilizes a low frequency but high volume controlled expiration, serving as breathing support to bridge patients from mechanical ventilation to spontaneous breathing (28, 29). With novel ventilation systems that enable full ventilation through a small-bore lumen, it is anticipated that the number of access-related temporary tracheostomies in TORS patients can be further lowered.

In this systematic review, postoperative hemorrhage was the most common complication associated with TORS, which was reported in 8 cases, followed by swallowing difficulties and aspiration, each accounting for 5 cases. With regard to TORS-related bleeding, arterial ligation of branches of the external carotid artery may help to reduce its incidence as reported in one study (30). Dysphagia and subsequent aspiration as mentioned were transient complications that resolved with time. In patients with ELPS performed, subcutaneous emphysema and aspiration pneumonia were the most common complications as reported in 18 and 15 patients, respectively. Postoperative bleeding was reported in 11 patients. Surgical emphysema has been a known complication of ELPS due to the manipulation of pharynx and airway, and it is usually benign and self-limiting that resolve in days. Nonetheless, no fatal consequence resulted.

Comparison of postoperative complications was made between TORS and ELPS, showing no statistical difference between postoperative hemorrhage, swallowing difficulty, and the need for permanent tracheostomy. Statistically significant complications were PEG feeding and aspiration pneumonia with the *p*-value of 0.03 and 0.01, respectively. TORS has demonstrated a higher risk of requiring PEG feeding when compared to ELPS. Yet, this could be accounted by the difference in the tumor staging or center preference in performing tracheostomy in which further randomized controlled trials may be required to prove the relationship between TORS and likelihood of PEG feeding.

Postoperative bleeding risk was compared between the 2 groups of patients; despite not being statistically significant, TORS has shown a slightly lower incidence rate of bleeding compared to ELPS. The upmost haemostatic function demonstrated by TORS could probably be accounted by the feasibility of using 2-man 4-hand surgery as in conventional open technique with the use of endoclip that is unlikely to be performed in ELPS.

Oncological outcomes between TORS and ELPS were not compared in this systematic review due to the variation in the follow-up period as reported in different studies, which made a direct comparison between different treatment modalities challenging.

Despite the promising oncological and functional outcomes concluded from these studies, TORS and ELPS are not without disadvantages and limitations. For TORS, monopolar cautery frequently created a wider and deeper incision when compared to laser incision while causing more thermal damage to surrounding tissues. It resulted in a higher risk of airway compromise when compared to transoral laser microsurgery, another emerging treatment approach for HPC (31). Although similar oncological outcomes were achieved, the 5- to 10-mm resection margins in TORS were wider than the 1- to 2-mm margins in ELPS (32). Other disadvantages of TORS were discussed above as postoperative complications, of which bleeding, transient swallowing dysfunction, and subsequent aspiration accounted for the majority.

Regarding the limitation of TORS, a few have been identified. Most of the current data supported the use of TORS only in early-stage HPC, whereas outcomes for advanced-stage disease were not available (33). In addition, due to the bulky robotic instruments applied in TORS, obtaining a good visualization and sufficient space for surgical procedures has always been a challenge (34). Thus, in most of the studies, patients with poor mouth opening, retrognathic mandible, brachygnathia, and trismus were often excluded due to an inadequate operating field (13, 35, 36). Yet, trismus is seen in patients with previous RT for head and neck cancers. These individuals hence will not be able to benefit from this MIS. Another criticism directed against the results of TORS is that most patients studied were offered adjuvant therapy depending on the pathological staging, implying that the promising oncological outcomes that concluded from the listed studies could not be attributed to TORS alone (15).

For ELPS, no clinically significant disadvantages have been identified, except that the application of ELPS was limited by the indications. The ELPS procedure currently is only indicated for superficial lesions, such as carcinoma *in situ* or early carcinoma without muscular invasion (33). The anatomical site of tumors is another major limitation since ELPS does not apply to all laryngeal cancer. Careful selection and preoperative investigations were required before offering ELPS to patients with HPC.

Several limitations were identified in this systematic review. First of all, most of the studies included in this review are of small sample sizes and short follow-up time ranging from 1 to 5 years. Particularly for ELPS, the samples were small, from Japan alone, retrospective in nature and lacked a control group, making the analysis of postoperative complications and recurrence difficult. In addition, no recent or existing data on the oncological outcomes of conventional surgical approaches or chemoradiotherapy were published or used as study controls. Thus, a fair comparison of minimally invasive surgery against current treatment modalities could not be made. Furthermore, other popular endoscopic organ preservation surgeries such as TLM were not included, which have been reported to attain comparable oncological outcomes and fewer complications (8, 37, 38).

For future reviews, studies with longer follow-up periods and larger cohorts of patients should be included to facilitate comparisons of the oncological outcomes of TORS and ELPS for HPC with other treatment modalities. Other emerging organ preservation treatments can also be included to complement the discussions.

## Conclusions

Both TORS and ELPS are safe and reliable techniques with acceptable oncological and functional outcomes in the resection of HPC. Numerous clinical studies have exhibited improved swallowing function compared with conventional approaches. With further studies and clinical trials, TORS or ELPS can potentially improve the management of cancers of the hypopharynx.

## Data Availability Statement

The original contributions presented in the study are included in the article/supplementary material, further inquiries can be directed to the corresponding author/s.

## Author Contributions

DY, JC, KL, and RL contributed to conception and design of the study. DY and JC drafted the exclusion and inclusion criteria and selected the studies for final inclusion in this systematic review. DY, JC, and KL screened the studies. DY and KL organized the database and performed the statistical analysis. JC and DY screened the statistics and contributed to the data presentation. KL wrote the first draft of the manuscript. DY, JC, KL, RL, BL, and C-CW wrote sections of the manuscript. All authors contributed to manuscript revision, read, and approved the submitted version.

## Conflict of Interest

The authors declare that the research was conducted in the absence of any commercial or financial relationships that could be construed as a potential conflict of interest.

## Publisher's Note

All claims expressed in this article are solely those of the authors and do not necessarily represent those of their affiliated organizations, or those of the publisher, the editors and the reviewers. Any product that may be evaluated in this article, or claim that may be made by its manufacturer, is not guaranteed or endorsed by the publisher.
